# Profiles of Acetylation Regulation Genes Contribute to Malignant Progression and Have a Clinical Prognostic Impact on Liver Cancer

**DOI:** 10.1155/2022/1724301

**Published:** 2022-09-10

**Authors:** Wei Zhu, Xiaofen Zhang, Mengli Yu, Yu Zhang, Shaowei Li, Chaohui Yu

**Affiliations:** ^1^Department of Gastroenterology, The First Affiliated Hospital, Zhejiang University School of Medicine, Hangzhou, Zhejiang 310000, China; ^2^Department of Gastroenterology, Taizhou Hospital of Zhejiang Province, Zhejiang University, Linhai, Zhejiang 317000, China

## Abstract

**Background:**

Several studies have demonstrated that acetylation was involved in the process of liver cancer. This study aimed to establish an effective predictive prognostic model using acetylation regulation genes in liver cancer.

**Methods:**

Two datasets were downloaded from the Cancer Genome Atlas (TCGA) database and International Cancer Genome Consortium (ICGC) database. Differentially expressed acetylation regulation genes were identified in the TCGA-LIHC dataset, and then, Gene Ontology (GO) functional annotation analysis was used to investigate the molecular mechanism. After grouping the patients into clusters based on consensus clustering, we explored the correlation between clusters and clinical characteristics. A risk model was constructed by the least absolute shrinkage and selection operator (LASSO) regression analysis to calculate the risk score. Patients were divided into high-risk and low-risk groups according to the risk score using the acetylation regulation genes. Data downloaded from LIRI-JP were used for external validation. Univariate and multivariate Cox regressions were performed to identify independent risk factors. A prognostic nomogram was constructed according to the TCGA-LIHC dataset. The effect of HDAC11 expression on the proliferation and migration of liver cancer was detected by the CCK-8 method and cell scratch test, respectively.

**Results:**

Eleven of 29 acetylation regulation genes were identified as upregulated differentially expressed genes. Go enrichment analysis showed that they were involved in “protein and histone deacylation and deacetylation.” Patients were categorized into two clusters according to the expression of 29 acetylation regulation genes. Compared with cluster 2, cluster 1 correlated with shorter overall survival (OS) and higher expression. Stage, T stage, grade, gender, age, and follow-up state were significantly different between two clusters. Pathways involved in DNA repair were significantly enriched in cluster 1. The risk score was calculated by HDAC1, HDAC2, HDAC4, HDAC11, HAT1, and SIRT6. Patients in the high-risk group had a worse prognosis in both datasets. Risk score was not only an independent prognostic marker but could also predict the clinicopathological features of liver cancer. A nomogram containing risk score, T stage, and M stage was built to predict overall survival. After transfection with HDAC11 overexpression plasmid, the proliferation ability of HepG2 cells increased, while the migration ability had no change.

**Conclusions:**

Our findings suggested that acetylation regulation genes contribute to malignant progression and have a clinical prognostic impact on liver cancer.

## 1. Introduction

Primary liver cancer comprises hepatocellular carcinoma, intrahepatic cholangiocarcinoma, and other rare tumors [1]. The prognosis for liver cancer is poor [2]. Neither current ablation therapies nor chemotherapy is appreciably effective in improving outcomes of this devastating disease [2]. Therefore, it is a priority for us to identify high-risk patients and ascertain novel therapeutic targets as well as effective treatment options for this disease.

Since being proposed, considerable evidence supports the pivotal role of cancer stem-like cells (CSCs) in pathological self-renewal, drug resistance, and cellular heterogeneity of cancer. Meanwhile, the epigenetic aberration in normal developmental processes is a key driver of CSC-like properties [3, 4]. Epigenetic modifications, including DNA methylation, histone modification, nucleosome remodeling, and RNA-mediated targeting, regulate many biological processes that are fundamental to the genesis of cancer [5]. Acetylation is one of the most important protein modifications, which contributes to several chromatin-dependent processes, such as DNA replication, damage and repair, transcriptional activation, cell cycle, and gene regulation [3], and in turn modulates cellular activities like proliferation, differentiation, and migration [6]. The dynamic process is regulated by the balance between histone acetyltransferases (HATs) and deacetylases (HDACs) [6]. Previous studies have revealed that several members in HATs and HDACs are involved in liver cancer progression [7–9], growth [10], proliferation, migration [7, 11], and chemoresistance [12]. It has been observed that in different databases, several HDACs increase with a strong expression variant in liver cancer compared to adjacent normal tissues [13]. Also, some HDACs inhibitor can activate apoptosis in liver cancer [14] and has been approved as a potential treatment for early-stage liver cancer in animal trials [15] and cell experiments [13]. However, most previous studies focus on the effect of a single gene, while the precise role of each HATs and HDACs in regulating tumorigenesis remains unclear. Because of the overlap of the target genes of acetylation regulation genes and the existence of joint regulation by the same factor [16], the profile of cellular acetylation status and gene expression level is of great importance to exploring new treatments and assessing the potential benefit of the existing inhibitors.

Therefore, through this novel study, we attempt to establish an effective predictive prognostic model using acetylation regulation genes in liver cancer. We detected 11 different expression genes (DEGs) between liver tumor tissue and adjacent normal tissues by systematically analyzing the expression of 29 acetylation regulation genes in TCGA-LIHC cohort. Gene Ontology (GO) functional annotation analysis was performed using these 11 DEGs to explore the underlying molecular mechanism. Next, we divided patients into two clusters according to 29 acetylation regulation genes' expression and confirmed the relation between clustering and malignant progression. Gene set enrichment analysis (GSEA) was used to find various Kyoto Encyclopedia of Genes and Genomes (KEGG) pathways that might explain the mechanism of different prognoses enriched in different clusters. Besides, we built a risk model including HDAC1, HDAC2, HDAC4, HDAC11, HAT1, and SIRT6 through the least absolute shrinkage and selection operator (LASSO) regression analysis. Patients included in the high-risk group had a worse prognosis. The prediction value was validated in TCGA-LIHC and LIRI-JP datasets using Kaplan-Meier curve and receiver operating curve (ROC). Association between risk score and clinical characteristics was also investigated. Univariate and multivariate Cox regressions testified risk score as an independent prognostic risk factor. The nomogram consisting of risk score, T, and M, was built to predict overall survival. Cell experiment confirmed overexpression of HDAC11 accelerated cell proliferation. Therefore, we put forward that acetylation regulation genes may contribute to malignant progression, and these six genes have a clinical prognostic impact on liver cancer.

We present the following article in accordance with the MDAR reporting checklist.

## 2. Materials and Methods

### 2.1. Data Acquisition

In this study, the RNA-seq transcriptome data of 374 tumor tissues and 50 adjacent normal tissues and the clinical information of 348 patients in the TCGA-LIHC dataset were downloaded from the Cancer Genome Atlas (TCGA) data portal (https://portal.gdc.cancer.gov/). LIRI-JP dataset was obtained from International Cancer Genome Consortium (ICGC) data portal (https://dcc.icgc.org/), containing the RNA-seq transcriptome data of 202 normal tissues and 243 liver tumor tissues and the clinical information of 232 patients. RNA-sequencing data were normalized as fragments per kilobase million (FPKM). The extracted clinical information, such as gender, age, grade, stage, T stage, M stage, N stage, follow-up time (futime), and follow-up state (fustate), was used in the following analysis.

### 2.2. Acetylation Regulation Genes and DEGs Identification

There were 29 well-studied genes in acetyltransferases and deacetylases families included as target genes in our study [6, 17], listed as follows: HDAC1-11 [8, 15, 18–20], SIRT1-7 [7, 8, 21–23], KAT2A [24], KAT2B, KAT5, KAT6A, KAT6B, KAT7-8, HAT1, CREBBP [7], EP300 [18], and NAA60. We extracted the mRNA expression matrix of these genes and the clinical information of patients for subsequent bioinformatic analysis. The missing data was processed by list deletion. The overall survival (OS) was defined as the interval from the date of diagnosis to the date of death.

To investigate the DEGs of acetylation regulation genes between liver cancer and adjacent normal tissue, we used the “Limma” package to analyze the expression of 29 genes in samples of TCGA-LIHC. After log_2_-transformed, these genes expression was compared between normal tissue and tumor tissue by using the Wilcoxon test. After calculating the mean value and log_2_FC (fold change) (log_2_FC = log_2_ (tumor mean value)/(normal mean value)) of each target gene, the genes with *p* < 0.05 and |log_2_*FC*| ≥ 1 were considered as DEGs and the result was visualized by a vioplot.

### 2.3. Bioinformatic Analysis

#### 2.3.1. GO Functional Annotation Analysis

Functional analyses of GO pathway were conducted to determine the major pathways regulated by these DEGs, using several packages such as “clusterProfiler,” “enrichplot,” “ggplot2,” and “GOplot.” The *p* value cutoff and *q* value cutoff were equal to 0.05. The top results were presented by barplot and GO circle plot.

#### 2.3.2. Identification of Consensus Clusters

The correlation analysis was conducted by “corrplot” package to identify the correlation among 29 target genes' expression in liver cancer. And then, we used the “ConsensusClusterPlus” package to group liver cancer patients into different clusters and used principal component analysis (PCA) to verify the grouping results.

Next, we drew a Kaplan-Meier survival curve of patients in different clusters using the “survival” package, and the correlation between clinical characteristics and clusters was conducted to determine the relationship between clustering and malignant progression. GSEA was used to find out various KEGG pathways enriched in different clusters.

#### 2.3.3. Identification of Risk Model and External Validation

We performed a univariate Cox regression analysis of the target genes' expression matrix to find genes related to a worse prognosis. Then, we used LASSO regression to generate a risk model to delete redundant genes predicting clinical prognosis and used ROC curve to determine the cutoff value. Patients were divided into high-risk and low-risk groups accordingly. Meanwhile, according to the risk model formula originating from the TCGA-LIHC dataset, the risk scores of patients in the LIRI-JP dataset were calculated. Besides, patients were rated into high-risk and low-risk groups using the cutoff determined from their own ROC. The *p* value of the Kaplan-Meier survival curve and AUC of ROC were used to evaluate the prediction value in both datasets. Finally, univariate Cox regression analysis and multivariate Cox stepwise regression were used to identify independent prognostic risk factors in the TCGA-LIHC dataset.

#### 2.3.4. Predictive Nomogram Construction and Evaluation

Variables identified in multivariate Cox stepwise regression were used to construct a predictive nomogram via “rms” package in the TCGA-LIHC dataset. The fit model was built by the function of “cph.” Calibration curve and Harrell's concordance index (C-index) were used to evaluate nomogram discrimination. Both of them were calculated using a bootstrap method with 1000 resamples. Sixty-five patients per group compared the concordance between nomogram predicted OS and observed OS at different time points. Harrell's C-index was calculated by internal sampling validation. The mean value of C-index evaluated the nomogram discrimination.

### 2.4. The Effect of HDAC11 on Liver Cancer

#### 2.4.1. Cell Culture

Human hepatoblastoma cell line HepG2 was obtained from the Chinese Academy of Science (Shanghai, China). Cells were cultured in DMEM supplemented with 10% FBS and 1% penicillin/streptomycin in 5% CO_2_ at 37°C.

#### 2.4.2. Transfection

PcDNA3.1-NC and pcDNA3.1-hdac11 plasmids were provided by GENEray (Shanghai, China). Cells were harvested after >24 h transfection in HepG2 using Lipofectamine 3000 (Invitrogen, USA).

#### 2.4.3. Western Blot

RIPA lysis buffer (Fdbio, China) with protease inhibitors was used to obtain total protein extraction. Equal amounts of protein were separated by 12% SDS-PAGE followed by transfer to PVDF membranes (Millipore, USA). Primary antibodies used included mouse anti-HDAC11 (Santa Cruz, USA) and anti-GAPDH (CST, USA). Signal was developed using an ECL Kit (Fdbio, China) with detection on a ChemiScope system (Clinx Science, China).

#### 2.4.4. CCK-8 Assay

Cell survival rates were estimated by the CCK-8 assay (APExBIO, USA). After transfection, approximately 10^3^ cells were seeded in 96-well plates with 100 *μ*l medium for each well. At 0, 24, 48, 72, and 96 h, the original medium was removed, and a medium supplemented with 10% CCK-8 solution was added and incubated for 2 h. The absorbance at 450 nm was measured. The proliferation curve was plotted according to log (ODt1/OD0h, 2).

#### 2.4.5. Cell Scratch Test

A single scratch was made using a sterile 10*-μ*l pipette tip, while the cells seeded in a 6-well plate reached a confluent state. Then, cells were washed with PBS 2 times, and the medium was replaced by FBS-free DMEM and incubated for another 24 h. Images of the scratches were captured at 0 and 24 h with Olympus IX73. The width of the scratch was analyzed using the Image Pro Plus software.

### 2.5. Statistical Analysis

The statistical and bioinformatic analysis was conducted by using R software (version 4.0.3), and related packages (“limma,” “ggplot2,” “survival,” “survminer,” “forestplot,” and “glmnet”) were needed to install and load during analysis. GSEA was conducted by using GSEA software (version 4.1.0). Wilcoxon tests were used to compare the expression level between tumor tissues and adjacent normal tissues, and Fisher tests were used to compare the differential distribution of age, gender, stage, T status, M status, and N status in different groups.

After constructing the risk model, we obtained the optimal cutoff value depending on ROC curve and divided the patients into high-risk and low-risk groups. The significant prognostic risk factor was identified by univariate Cox regression analysis and multivariate Cox stepwise regression analysis (*p* < 0.05). All cell experimental results were independently repeated at least three times. *T*-test or one-way ANOVA test was used to analyze proliferation and migration ability. *p* < 0.05 was statistically significant.

## 3. Results

### 3.1. The Distinction of Acetylation Regulation Genes in Liver Cancer and Normal Tissue

**Figure 1:**
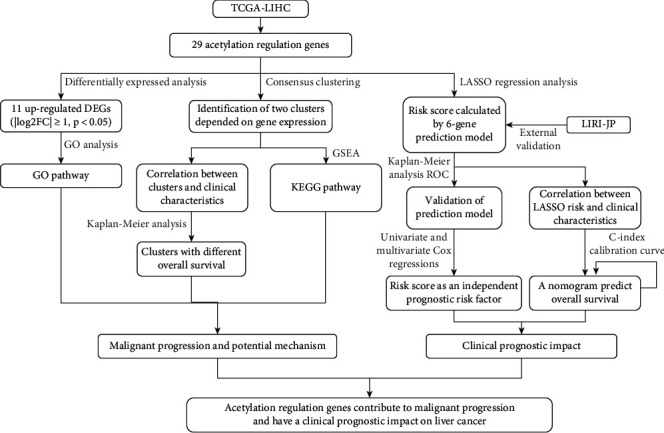
Flowchart presenting the process in this study.

**Figure 2:**
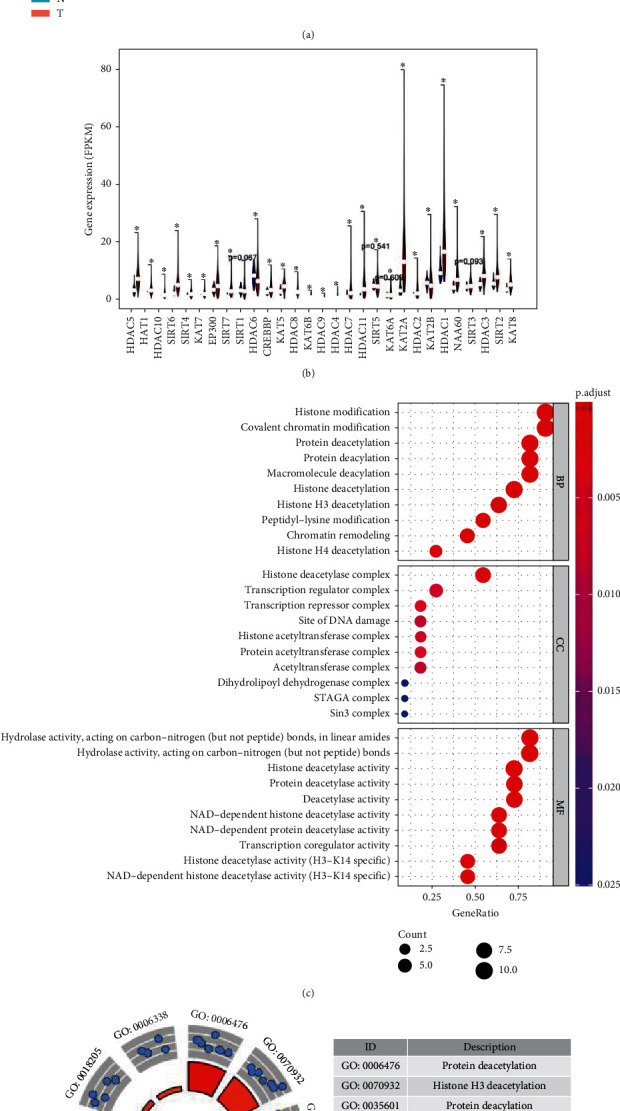
The profiles of acetylation regulation genes in liver cancer. (a) The heat map of 29 acetylation regulation genes in liver cancer. Red presents upregulated gene, and green presents downregulated gene. Lake blue represents adjacent normal tissue, and pink represents liver tumor tissue. (b) Vioplot visualizes the differential expression of acetylation regulation genes between liver cancer tumor tissue and adjacent normal tissue. Blue is adjacent normal tissue, and red is liver tumor tissue. (c) Enrichment pathway of 11 DEGs in the GO bubble plot. The larger bubble and red color indicated the more significant enrichment process. (d) Enrichment pathways in the GO circle plot. The inner circle indicates *Z*-score, and the red color represents the significant enrichment. The outer circle indicates the various pathways, and blue dots indicate upregulated genes.^∗^*p* < 0.05, ^∗∗^*p* < 0.01, and ^∗∗∗^*p* < 0.001.

This study is performed according to the flowchart in Figure 1. Considering the importance of each acetylation regulation gene in tumorigenesis and the development of liver cancer, we compared the expression of 29 genes in 374 tumor tissues and 50 adjacent normal tissues (Figure 2(a)). The heat map and vioplot showed that there were 25 genes (Figures 2(a) and 2(b)), the expression levels of which were significantly different between tumor tissue and normal tissue. Among them, the expression of HDAC6 and KAT2B decreased in tumor tissue, while the rest increased. HDAC1, HDAC4, HDAC5, HDAC7, HDAC10, HDAC11, KAT2A, KAT7, SIRT4, SIRT6, and SIRT7 were upregulated and identified as significant DEGs between normal and tumor tissues (Table 1). Among them, HDAC7, HDAC4, SIRT7, KAT2A, and HDAC11 were the top five most upregulated genes, indicating that the expression of deacetylation-related genes played a dominant role in liver cancer compared with acetylation-related genes.

GO analysis was conducted to analyze the function of these 11 genes in tumorigenesis and progression of liver cancer. The bubble plot (Figure 2(c)) showed the result listed in descending order by gene ratio in each pathway. In terms of biological process (BP), pathways such as “histone modification,” “covalent chromatin modification,” “protein deacetylation,” and “protein deacylation” increased. Besides, “histone deacetylase complex” and “transcription regulator complex” increased as cellular component (CC), and the molecular function (MF) of “hydrolase activity,” “histone deacetylase activity,” and “protein deacetylase activity” was upregulated in tumor tissue (Figures 2(c) and 2(d)). The top 5 results in BP, CC, and MF in ascending order by *p* value are listed in Supplementary Table S1.

### 3.2. Consensus Clustering of Acetylation Regulation Genes Identified Two Clusters of Liver Cancer

**Figure 3:**
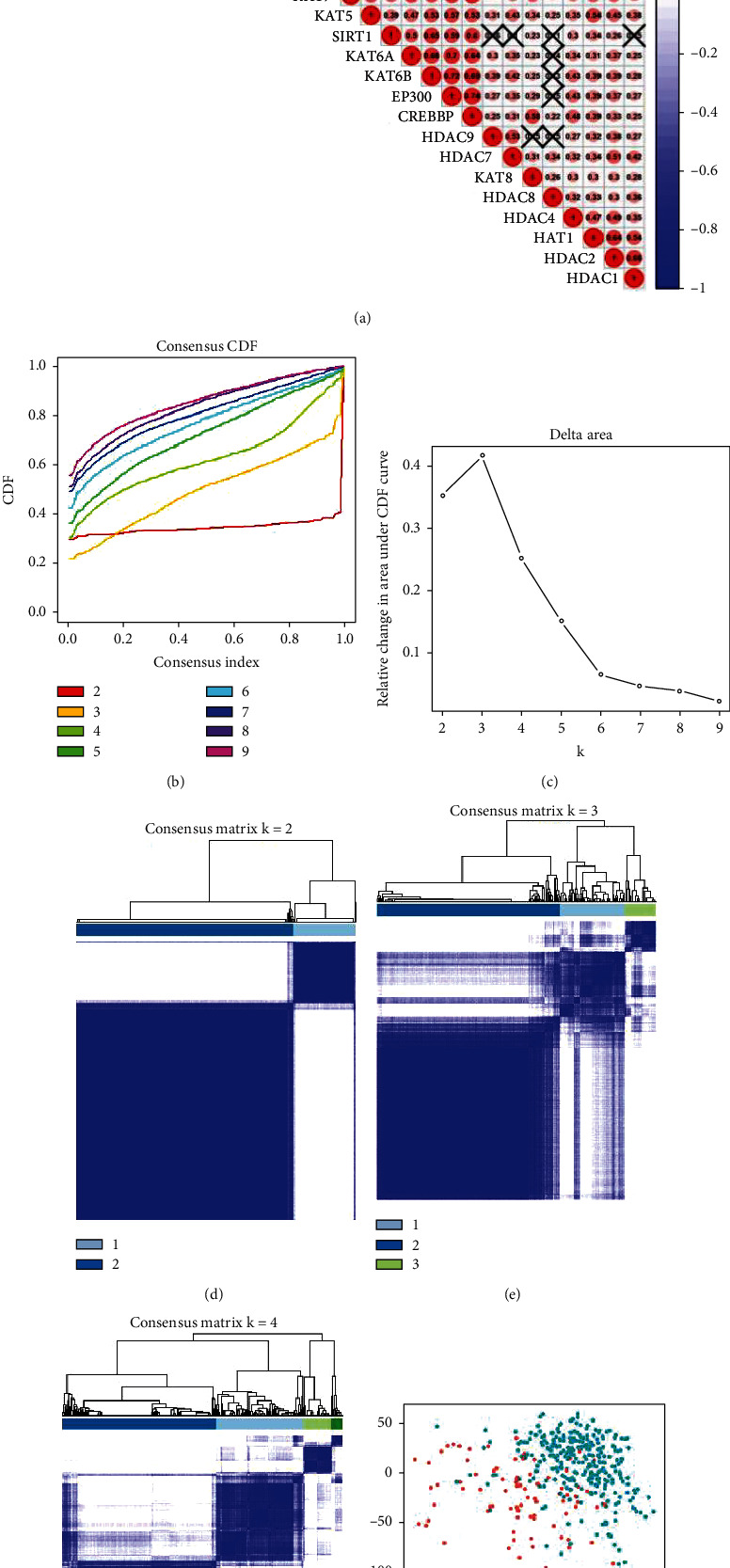
Identification of consensus clusters by acetylation regulation genes. (a) Correlation analysis of 29 acetylation regulation genes in liver cancer. Red is positive correlation, blue is negative correlation, and × in each box is no correlation. The number in each box represents correlation coefficient between two cross genes. (b) Consensus clustering CDF for *k* = 2–9. (c) Relative change in area under CDF curve for *k* = 2–9. (d–f) Consensus clustering matrix for *k* = 2, *k* = 3, and *k* = 4. (g) PCA of the total RNA expression profile. Patients in cluster 1 are marked with red, and those in cluster 2 are marked with lake blue.

We supposed the expression of acetylation regulation genes might correlate to different clinical characteristics in liver cancer. Firstly, we investigated the correlation analysis of 29 acetylation regulation genes in liver cancer.  Figure 3(a) shows that a large proportion of target genes were weakly to moderately correlated, which meant those genes could not be considered as a single independent variable during subsequent analysis. Next, we used “ConsensusClusterPlus” package to group patients into different clusters. The fit *k* value needed to meet three criteria: (1) The ideal *k* value should be chosen as the cumulative distribution function (CDF) reaching a maximum approximation, (2) the number of patients in each cluster should not be too small, and (3) the gene expression should be a strong correlation in each cluster, while the correlation between clusters should be as weak as possible. Based on the results of Figures 3(b) and 3(c), *k* = 4 seems to have a minor cumulative distribution function (CDF) increment, but taking the number of patients in each cluster and the correlation among different clusters into consideration (Figures 3(d)–3(f)), *k* = 2 was more appropriate in our study. To confirm whether our classification was correct, we performed PCA using the whole RNA-seq data and found that patients in respective clusters could gather together (Figure 3(g)). This result indicated that the classification of liver cancer patients into two clusters by target genes was correct.

### 3.3. Correlation between Clusters and Clinical Characteristics and Potential Mechanisms

**Figure 4:**
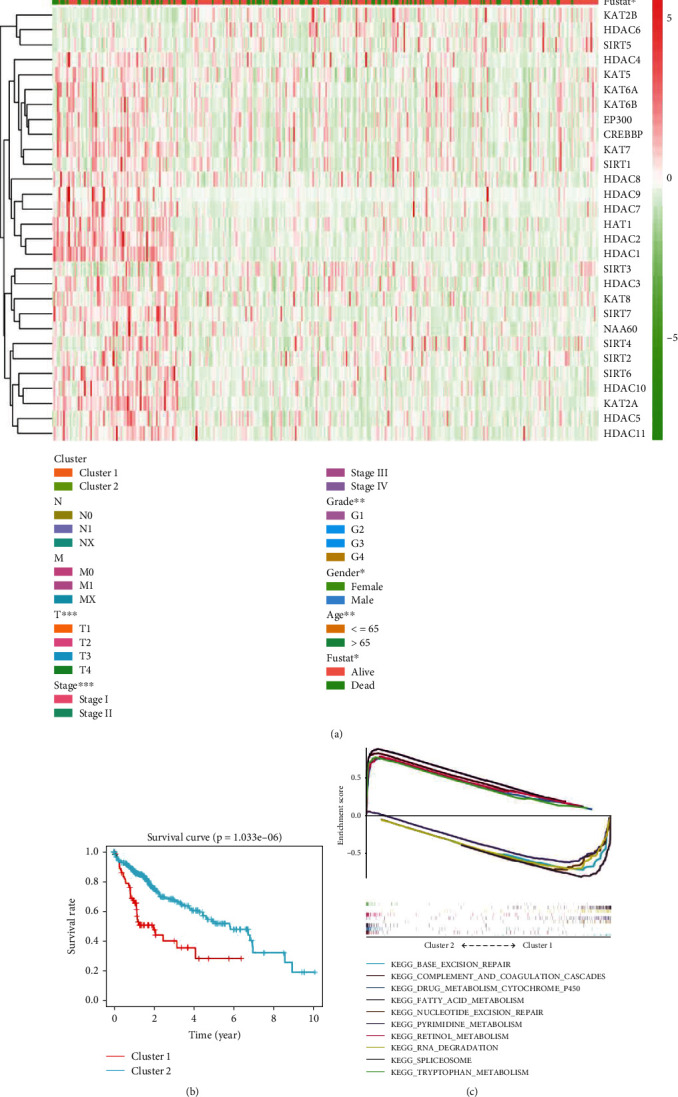
Different clinical characteristics of liver cancer in cluster 1 and cluster 2. The consensus expression acetylation regulation genes define cluster 1 and 2. (a) The heat map of clinical characteristics and gene expression in two clusters. (b) Kaplan-Meier survival curves for 374 liver cancer patients. Liver cancer patients in cluster 1 are marked with red, and those in cluster 2 are marked with lake blue. (c) GSEA of the genes in 2 clusters using the KEGG pathway. ^∗^*p* < 0.05, ^∗∗^*p* < 0.01, and ^∗∗∗^*p* < 0.001.

After grouping the patients, we investigated the correlation between clusters and clinical features.  Figure 4(a) shows that stage (stage I-IV), T stage (T1-T4), grade (G1-G4), gender (male/female), age (≤65, >65), and follow-up state (alive/dead) were significantly different in two clusters. Besides, we found that most of the target genes had higher expression in cluster 1. The OS of patients in cluster 2 was much longer than that in cluster 1 (Figure 4(b)), and the difference of 3-year survival rates was statistically significant (cluster 1 vs cluster 2 =40.5% vs 67.6%). In other words, the expression profile of acetylation regulation genes was expected to predict prognosis in liver cancer.

To investigate the potential mechanisms caused this survival difference, we used GSEA to find out different KEGG pathway between two clusters. The top 5 regulated KEGG pathways with the highest normalized enrichment score in respective clusters are listed in Figure 4(c). The pathways such as “complement and coagulation cascades,” “retinol metabolism,” “drug metabolism cytochrome p450,” “fatty acid metabolism,” and “tryptophan metabolism” were upregulated in cluster 2. Meanwhile, “spliceosome,” “RNA degradation,” “pyrimidine metabolism,” “base excision repair,” and “nucleotide excision repair” were upregulated in cluster 1 (Figure 4(c)), which may endow tumor cells stronger vitality.

### 3.4. Prognostic Value of Risk Model Consisting of Acetylation Regulation Genes

**Figure 5:**
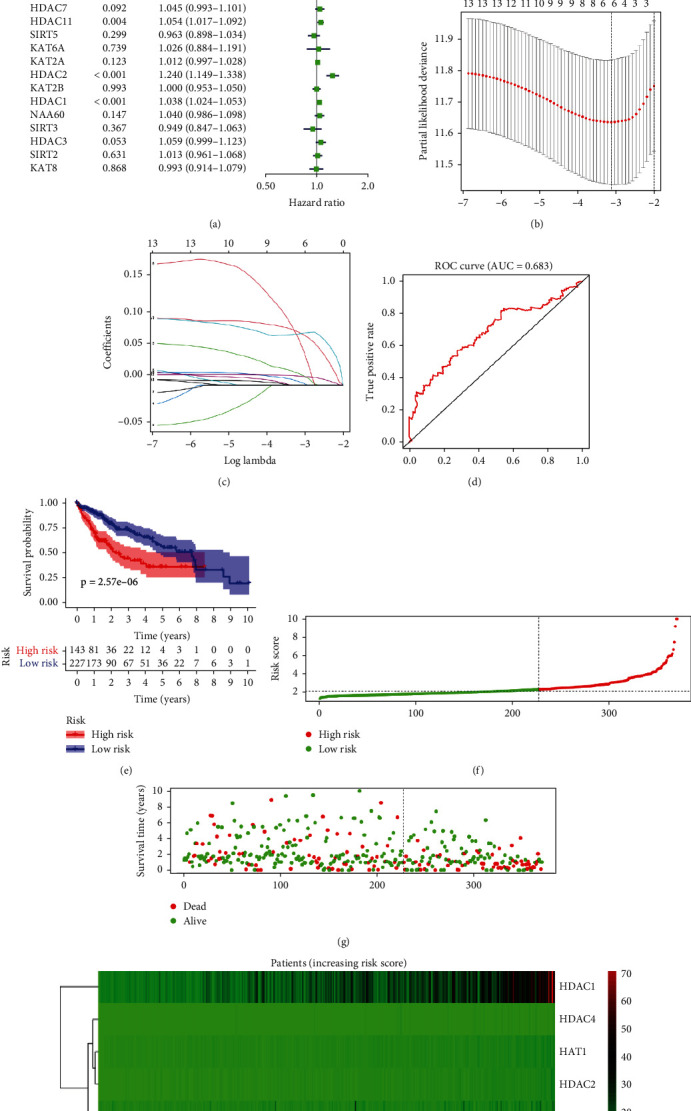
Risk signature of acetylation regulation genes. (a) Target genes correlate with worse clinical prognosis in forestplot. The HR and 95% CI are calculated by univariate Cox regression. (b, c) The optimal gene numbers and coefficients are calculated using LASSO's multivariate Cox regression. (d) ROC shows the predictive efficiency of the risk signature in 3-year survival rates. (e) Based on the risk score, the Kaplan–Meier survival curves of 374 liver cancer patients in the high-risk and low-risk groups. (f–g) Risk score of each patient and the respective survival time. (h) The heat map of 6 genes included in the LASSO risk score model in two groups.

We performed a univariate Cox regression analysis to investigate better the prognostic role of acetylation regulation genes in liver cancer. The results indicated that HDAC1, HDAC2, HDAC4, HDAC5, HDAC11, HAT1, SIRT6, SIRT7, and KAT7 were prognostic risk factors, with hazard ratios (HR) >1 and lower 95% confidence intervals (CI) >1 (Figure 5(a), Table 2). Thirteen genes (HDAC1, HDAC2, HDAC3, HDAC4, HDAC5, HDAC7, HDAC11, HAT1, SIRT6, SIRT7, KAT5, KAT7, and EP300) with *p* < 0.1 entered LASSO Cox regression algorithm to delete redundant genes and constructed a risk model. Six genes were selected based on the minimum criteria, and the coefficients obtained from the LASSO algorithm were used to calculate the risk score for every patient included in our study (Figures 5(b) and 5(c)). The risk score was calculated as follows:

Risk score = exp {[(Expression value of HAT1) ×0.0706633890112458] + [(Expression value of SIRT6) ×0.0148275359702248] + [(Expression value of HDAC4) ×0.0573493877297365] + [(Expression value of HDAC11) ×0.00408455809173769] + [(Expression value of HDAC2) ×0.0699244618291594] + [(Expression value of HDAC1) ×0.0123873924505771]}.

“SurvivalROC” package was used to evaluate the performance of the risk model and to determine the optional cutoff when grouping the patients into high-risk and low-risk groups. According to 3-year survival rates, the AUC was equal to 0.683 (Figure 5(d)), and the cutoff was equal to 2.301581.

To confirm the grouping result, we drew Kaplan-Meier survival curves.  Figure 5(e) indicates that the patients divided into the high-risk group had a shorter OS (*p* < 0.05), especially when follow-up was less than 3 years. Besides, patients with higher LASSO risk scores tended to have shorter survival (Figures 5(f) and 5(g)). We also found that these 6 genes included in the LASSO algorithm had higher expression in the high-risk group (Figure 5(h)).

### 3.5. External Validation of Risk Model

**Figure 6:**
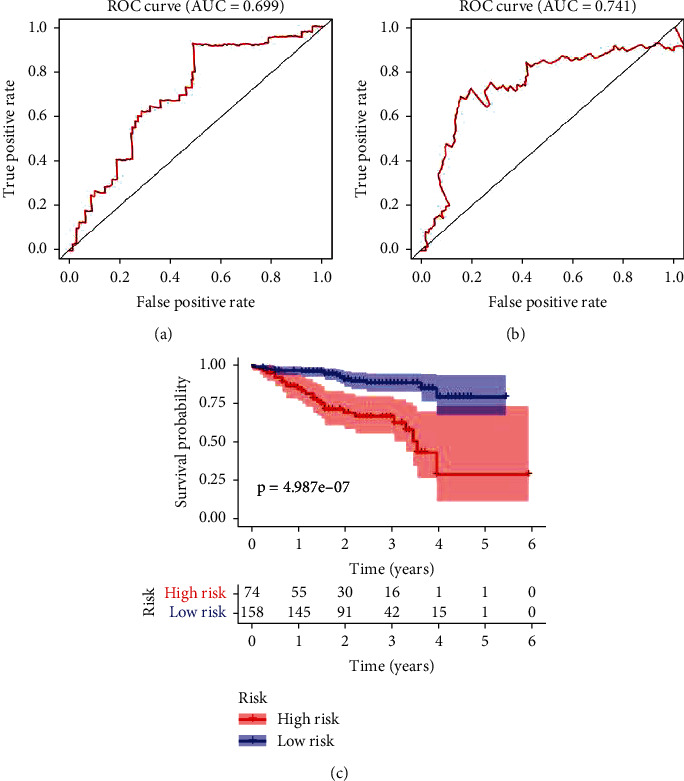
External validation of the risk model in the LIRI-JP dataset. (a–b) ROC shows the predictive efficiency of the risk model in 3-year (a) and 5-year (b) survival rates. (c) Based on the risk score, the Kaplan–Meier survival curves for 232 liver cancer patients in the high-risk and low-risk groups.

The mRNA expression matrix and clinical information of the six genes were extracted in the LIRI-JP dataset. The RNA-seq transcriptome data originated from 202 normal tissues and 243 liver tumor tissues in this dataset. After matching with clinical characteristics, a total of 232 patients were included in this study. The risk score of each patient was calculated using the formula mentioned above. According to the 3-year survival rates, the AUC was 0.699 (Figure 6(a)) with a threshold of 6.168746.

Furthermore, the AUC of 5-year survival rates was 0.741 (Figure 6(b)). One hundred and thirty-three patients were rated as high risk, while 99 patients were low risk. Kaplan–Meier survival curves showed that the OS in the high-risk group was much shorter than in the low-risk group (Figure 6(c)). These results confirmed that the risk score calculated by six genes effectively predicted liver cancer prognosis.

### 3.6. Associations between Risk and Clinicopathological Features in Liver Cancer and Independent Risk Factor Identification

**Figure 7:**
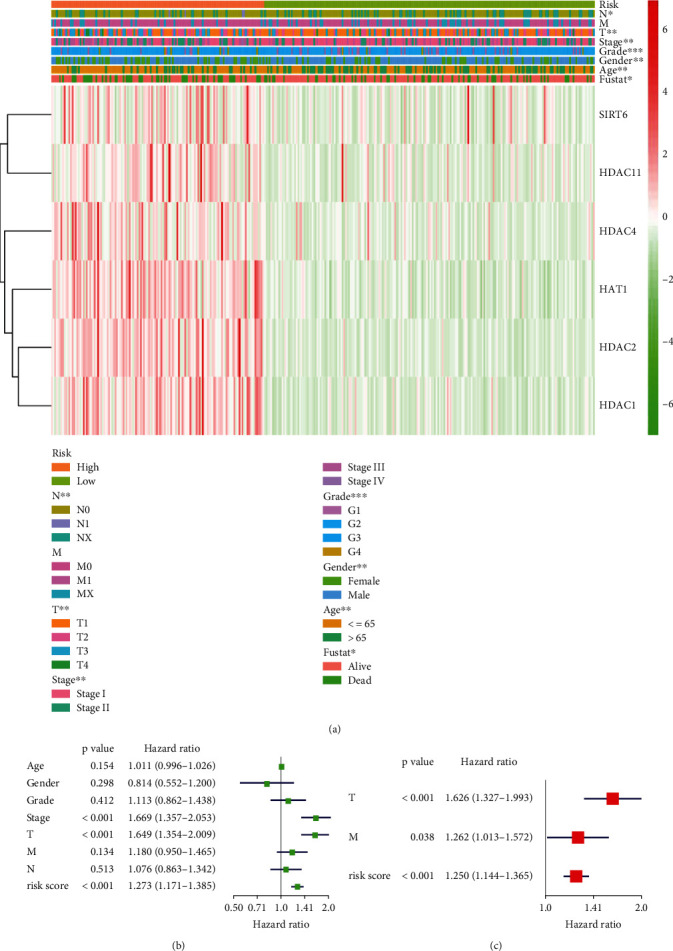
Association between the risk and clinicopathological features. (a) The heat map of clinicopathological features and gene expression in the high-risk and the low-risk groups. Univariate Cox regression analyses (b) and multivariate Cox regression analyses (c) are used to calculate HR to explore independent risk factors of liver cancer patients. ^∗^*p* < 0.05, ^∗∗^*p* < 0.01, and ^∗∗∗^*p* < 0.001.

The correlation between the risk model and clinical features was analyzed. We found that stage (stage I-IV), T stage (T1-T4), N stage (N0/N1/NX), grade (G1-G4), gender (male/female), age (≤65, >65), and follow-up state (alive/dead) were significantly differential distribution in high-risk and low-risk groups (Figure 7(a)).

Next, we used age, gender, grade, stage, T stage, M stage, N stage, and risk score in a univariate Cox regression analysis (Figure 7(b)). Three variables stage, T stage, and risk score with *p* < 0.05 were identified as risk factors. Furthermore, Cox regression stepwise analysis was used to build a fit model to predict prognosis with independent risk factors. Eventually, risk score, T, and M were included as independent prognostic risk factors in our study (*p* < 0.05) (Figure 7(c)).

### 3.7. Predictive Nomogram and Evaluation

**Figure 8:**
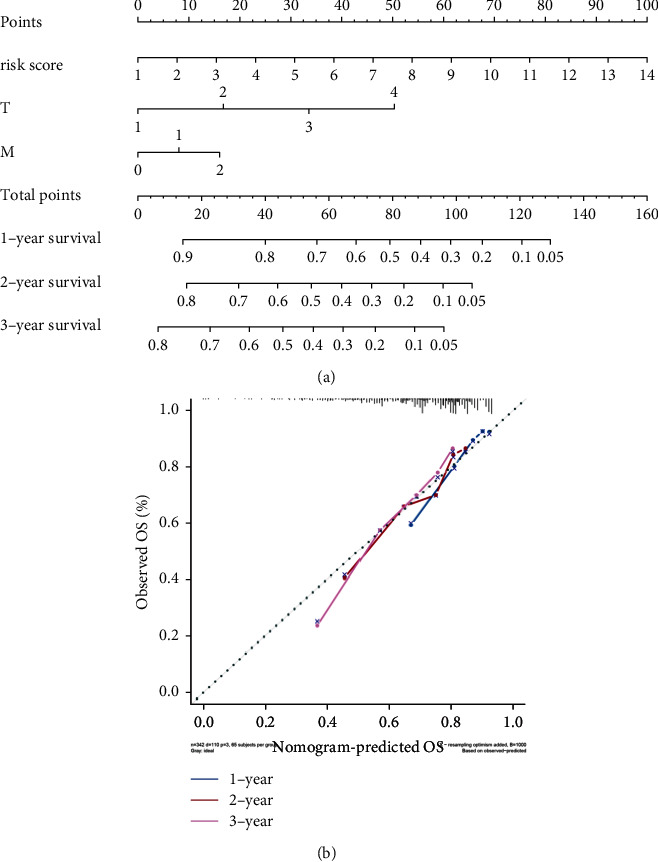
Validation of the predictive nomogram discrimination in the TCGA-LIHC dataset. (a) A prognostic nomogram predicts liver cancer's 1-year, 2-year, and 3-year OS. (b) The calibration curve for validation of the predictive nomogram.

The TCGA-LIHC dataset was used to construct the predictive nomogram. Independent risk factors identified by stepwise COX regression analysis, such as risk score, T, and M, were contained in predicting 1-year, 2-year, and 3-year OS (Figure 8(a)). Total points calculated by respective points based on risk score, T, and M were used to predict corresponding 1-year, 2-year, and 3-year OS. Calibration curves showed the ratio of nomogram predicted OS and observed OS always fluctuated around 1 (Figure 8(b)), indicating that the nomogram performed well. C-index was calculated by the internal bootstrap method with 1,000 resamples. The mean of C-index was 0.672.

### 3.8. HDAC11 Upregulated Cell Proliferation in HepG2

**Figure 9:**
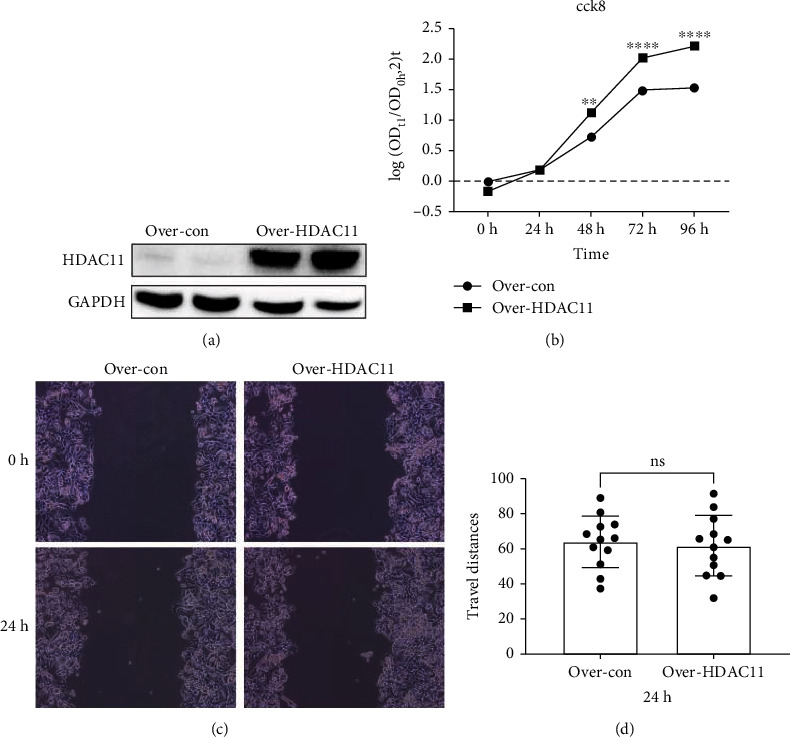
The effect of HDAC11 in liver cancer. HepG2 cell transfected with over-con and over-HDAC11 plasmids. (a) Expression of HDAC11 by western blot of protein extracts obtained from 24 h post-transfection. (b) Proliferation curve plots according to the result of the CCK-8 array. (c–d) The result of the cell scratch test and quantitative analysis.

CCK-8 proliferation curve indicated that the proliferation rate of HepG2 cells was significantly accelerated after transfection with HDAC11 plasmid compared with the control group (Figures 9(a) and 9(b)). After 48 h, the cell densities were much higher in the over-HDAC11 group compared with the over-con group (Figure 9(b)). The cell scratch test result showed no difference in the travel distance between the two groups (Figures 9(c) and 9(d)). Therefore, a high level of HDAC11 could upregulate cell proliferation ability.

## 4. Discussion

As the second leading cause of cancer-related death and a major public health challenge worldwide, the burden of liver cancer is increasing globally [1]. Liver cancer patients' outcomes are improved due to the optimization of individual treatment strategies and the development more complex therapeutic modalities [2].

Acetylation, considered one of the most critical protein modifications, plays an essential role in tumorigenesis and tumor growth, proliferation, migration, and chemoresistance in various cancer. Previous studies have demonstrated that acetylation and deacetylation influence the plasticity of chromatin structure by changing the electrical property of acetylated sites of histone and improving the stability of many nonhistone proteins by covering ubiquitination sites [16]. Protein acetylation modification regulated by HATs and HDACs exhibits different biological effects, leading to promotion [25] or suppression [26] in liver cancer, depending on the effect of protein. Even the same acetylation regulation gene can cause the opposite effect in liver cancer through regulating different target proteins [25, 26], let alone the genes in the same family [27, 28]. Considering these complex situations, integration of expression profiles of known acetylation regulation genes, which influence malignant progression and clinical prognostic in liver cancer, has great value and necessity. This study aimed to clarify the effect of acetylation regulation genes on liver cancer and attempted to construct a predictive prognostic model.

Twenty-five of 29 target genes had significant differences between tumor and normal tissues, and 11 were identified as DEGs. Nine of 11 DEGs were HDACs, including HDAC1, HDAC4, HDAC5, HDAC7, HDAC10, HDAC11, SIRT4, SIRT6, and SIRT7, and only KAT2A, and KAT7 in HATs were DEGs. GO analysis showed the activity of “protein deacetylation,” “histone H3 deacetylation,” “protein deacylation,” “macromolecule deacylation,” and “histone deacetylation” upregulated in tumor tissue. Therefore, we hypothesized that deacetylation of important proteins played an important role in developing liver cancer.

To explore the relationship between acetylation regulation genes and prognosis in liver cancer, we identified two clusters of liver cancer by applying consensus clustering to 29 genes. We found that cluster 1 with higher expression of most target genes was correlated with a poorer prognosis. Stage (stage I-IV), T stage (T1-T4), grade (G1-G4), gender (male/female), age (≤65, >65), and follow-up state (alive/dead) were significantly different in two clusters. The KEGG pathways involved in “spliceosome,” “RNA degradation,” “pyrimidine metabolism,” “base excision repair,” and “nucleotide excision repair” were significantly enriched in cluster 1. It was genomic repair and stability that we supposed endowed the tumor cells with better survival and subsequently caused a worse prognosis.

Also, we constructed a risk model by LASSO regression analysis. The risk score was calculated using the expression value of 6 acetylation regulation genes. All patients were rated into the high-risk and the low-risk groups accordingly. The ROC AUC of the risk score to predict clinical prognosis was 0.683. Generally, a model with AUROC =68.3% was not an ideal perfect one. But considering the complexity of tumorigenesis and its multiple factors contributed to prognosis, we supposed the risk prediction model using gene expression level has specific values. Furthermore, we explored other significant risk factors in the following analysis. We found that stage (stage I-IV), T stage (T1-T4), N stage (N0/N1/NX), grade (G1-G4), gender (male/female), age (≤65, >65), and follow-up state (alive/dead) were significantly differential distribution in high-risk and low-risk groups. Information extracted from the LIRI-JP dataset validated the predicted value of this risk model. Stepwise Cox regression showed that risk score, T, and M were independent risk factors in predicting prognosis in liver cancer. Finally, we construct a nomogram including the above-mentioned three variables. The calibration curve and C-index confirmed that the nomogram performed well.

This study analyzed the relationship between acetylation regulation genes and the development of liver cancer in its entirety and explored the potential mechanisms. We supposed deacetylation of pivotal proteins might contribute to tumorigenesis and progression of liver cancer. Six acetylation regulation genes were identified as independent risk factors to calculate the risk score, which was included in the prognostic nomogram, as well as T and M. Furthermore, the calibration curve and C-index confirmed that the performance of the nomogram was reliable.

HDAC11 was the most upregulated differentially expressed acetylation regulation gene. The role of HDAC11 in liver cancer was identified by CCK-8 array and cell scratch test. The results suggested that overexpression of HDAC11 could promote cell proliferation rather than alter cell migration.

However, this study had several limitations. First, two datasets are included in our study with limited sample sizes. So, it is necessary to verify our conclusion in more external datasets. Second, different datasets' risk scores may vary depending on different sequencing platforms. The optimal cutoff and nomogram are required to be redetermined accordingly before clinical application. Moreover, the protein expression levels of 6 genes and the total level of protein acetylation remain unclear, which requires the exploration of further experimental studies.

## 5. Conclusions

Our study verified that acetylation regulation genes contribute to malignant progression and have a clinical prognostic impact on liver cancer. The risk score calculated by the expression value of HDAC1, HDAC2, HDAC4, HDAC11, HAT1, and SIRT6 was an independent risk factor for liver cancer. The nomogram composed of risk score, T stage, and M stage could effectively predict overall survival. Overexpression of HDAC11 enhanced cell proliferation in liver cancer. The findings of our study may provide a new treatment insight and target for liver cancer and affect medical decision-making for clinical practice.

## Figures and Tables

**Table 1:** Differentially expressed acetylation regulation genes.

Gene	MGE (normal)	MGE (tumor)	log2FC	*p* value
HDAC1	9.67	19.70	1.03	6.77E-19
HDAC10	0.82	1.85	1.18	8.46E-20
HDAC11	0.88	4.65	2.40	1.68E-27
HDAC4	0.24	0.77	1.66	9.14E-23
HDAC5	3.35	7.82	1.22	1.08E-21
HDAC7	1.20	3.17	1.41	7.39E-15
KAT2A	3.46	15.28	2.14	1.06E-27
KAT7	1.04	2.16	1.06	9.80E-20
SIRT4	1.05	2.14	1.04	1.18E-15
SIRT6	2.38	5.76	1.27	1.63E-23
SIRT7	1.11	3.53	1.66	7.04E-27

MGE: mean gene expression, presented by FPKM value.

**Table 2:** Univariate Cox regression analysis of acetylation regulation genes.

Gene	HR	95% CI	*p* value
HAT1	1.25	1.14-1.36	7.58E-07
HDAC1	1.04	1.02-1.05	2.92E-07
HDAC11	1.05	1.02-1.09	0.003937024
HDAC2	1.24	1.15-1.34	2.88E-08
HDAC4	1.66	1.29-2.13	7.32E-05
HDAC5	1.06	1.01-1.10	0.007920889
KAT7	1.21	1.03-1.42	0.019357053
SIRT6	1.08	1.03-1.13	0.003052138
SIRT7	1.09	1.02-1.17	0.008986678

HR: hazard ratios; 95% CI: 95% confidence intervals.

## Data Availability

Data sharing is not applicable to this article as no datasets were generated during the current study.

## Supplementary Materials

Supplementary Materials Supplementary table S1: It shows the GO enrichment analysis.
