# Supplementary material for: Profiles of Acetylation Regulation Genes Contribute to Malignant Progression and Have a Clinical Prognostic Impact on Liver Cancer
Source: Dis Markers. 2022 Sep 10;2022:1724301. doi: 10.1155/2022/1724301 (PMC9482539; doi:10.1155/2022/1724301)
Supplement: Supplementary Materials — Supplementary table S1: It shows the GO enrichment analysis. [file 1724301.f1.docx]

| Supplementary Table S1: GO enrichment analysis | | | | | | | |
| --- | --- | --- | --- | --- | --- | --- | --- |
|  | | ID | Description | p-Value | p.adjust | geneID |  |
| BP | GO:0006476 | | protein deacetylation | 9.3E-20 | 5.6E-17 | HDAC5/HDAC10/SIRT6/SIRT4/SIRT7/HDAC4/HDAC7/HDAC11/HDAC1 |  |
| BP | GO:0070932 | | histone H3 deacetylation | 1.5E-19 | 5.6E-17 | HDAC5/SIRT6/SIRT7/HDAC4/HDAC7/HDAC11/HDAC1 |  |
| BP | GO:0035601 | | protein deacylation | 2.3E-19 | 5.8E-17 | HDAC5/HDAC10/SIRT6/SIRT4/SIRT7/HDAC4/HDAC7/HDAC11/HDAC1 |  |
| BP | GO:0098732 | | macromolecule deacylation | 3.3E-19 | 6.1E-17 | HDAC5/HDAC10/SIRT6/SIRT4/SIRT7/HDAC4/HDAC7/HDAC11/HDAC1 |  |
| BP | GO:0016575 | | histone deacetylation | 1.1E-17 | 1.6E-15 | HDAC5/HDAC10/SIRT6/SIRT7/HDAC4/HDAC7/HDAC11/HDAC1 |  |
| CC | GO:0000118 | | histone deacetylase complex | 1.4E-12 | 6.2E-11 | HDAC5/HDAC10/HDAC4/HDAC7/HDAC11/HDAC1 |  |
| CC | GO:0017053 | | transcription repressor complex | 8.0E-4 | 0.009 | HDAC4/HDAC1 |  |
| CC | GO:0090734 | | site of DNA damage | 9.3E-4 | 0.009 | KAT7/SIRT7 |  |
| CC | GO:0000123 | | histone acetyltransferase complex | 9.5E-4 | 0.009 | KAT7/KAT2A |  |
| CC | GO:0031248 | | protein acetyltransferase complex | 0.001 | 0.009 | KAT7/KAT2A |  |
| MF | GO:0004407 | | histone deacetylase activity | 5.6E-22 | 2.1E-20 | HDAC5/HDAC10/SIRT6/SIRT7/HDAC4/HDAC7/HDAC11/HDAC1 |  |
| MF | GO:0033558 | | protein deacetylase activity | 8.1E-22 | 2.1E-20 | HDAC5/HDAC10/SIRT6/SIRT7/HDAC4/HDAC7/HDAC11/HDAC1 |  |
| MF | GO:0016811 | | hydrolase activity, acting on carbon-nitrogen (but not peptide) bonds, in linear amides | 4.8E-21 | 8.4E-20 | HDAC5/HDAC10/SIRT6/SIRT4/SIRT7/HDAC4/HDAC7/HDAC11/HDAC1 |  |
| MF | GO:0017136 | | NAD-dependent histone deacetylase activity | 1.5E-20 | 2.0E-19 | HDAC5/SIRT6/SIRT7/HDAC4/HDAC7/HDAC11/HDAC1 |  |
| MF | GO:0034979 | | NAD-dependent protein deacetylase activity | 2.7E-20 | 2.9E-19 | HDAC5/SIRT6/SIRT7/HDAC4/HDAC7/HDAC11/HDAC1 |  |
